# Quantum Mechanical Calculations of Redox Potentials of the Metal Clusters in Nitrogenase

**DOI:** 10.3390/molecules28010065

**Published:** 2022-12-21

**Authors:** Hao Jiang, Oskar K. G. Svensson, Ulf Ryde

**Affiliations:** Division of Theoretical Chemistry, Department of Chemistry, Lund University, P.O. Box 124, SE-221 00 Lund, Sweden

**Keywords:** nitrogenase, redox potential, formal oxidation states, FeMo cluster, P-cluster

## Abstract

We have calculated redox potentials of the two metal clusters in Mo-nitrogenase with quantum mechanical (QM) calculations. We employ an approach calibrated for iron–sulfur clusters with 1–4 Fe ions, involving QM-cluster calculations in continuum solvent and large QM systems (400–500 atoms), based on structures from combined QM and molecular mechanics (QM/MM) geometry optimisations. Calculations on the P-cluster show that we can reproduce the experimental redox potentials within 0.33 V. This is similar to the accuracy obtained for the smaller clusters, although two of the redox reactions involve also proton transfer. The calculated P^1+^/P^N^ redox potential is nearly the same independently of whether P^1+^ is protonated or deprotonated, explaining why redox titrations do not show any pH dependence. For the FeMo cluster, the calculations clearly show that the formal oxidation state of the cluster in the resting E_0_ state is ${\mathrm{Mo}}^{\mathrm{III}}{\mathrm{Fe}}_{3}^{\mathrm{II}}{\mathrm{Fe}}_{4}^{\mathrm{III}}$ , in agreement with previous experimental studies and QM calculations. Moreover, the redox potentials of the first five E_0_–E_4_ states are nearly constant, as is expected if the electrons are delivered by the same site (the P-cluster). However, the redox potentials are insensitive to the formal oxidation states of the Fe ion (i.e., whether the added protons bind to sulfide or Fe ions). Finally, we show that the later (E_4_–E_8_) states of the reaction mechanism have redox potential that are more positive (i.e., more exothermic) than that of the E_0_/E_1_ couple.

## 1. Introduction

Gaseous nitrogen (N_2_) is the main component of our atmosphere, but nitrogen is still a limiting element for plant life and a major ingredient in synthetic fertilisers. The reason for this is that the triple bond in N≡N is extremely strong and inert, making N_2_ unavailable for most plants [1,2,3]. Industrially, N_2_ is converted to NH_3_ by the Born–Haber process, which was invited in the early 20th century and is considered as a major cause of the human population explosion [1]. It requires high temperature and pressure.

N_2_ + 8 e^−^ + 8 H^+^ + 16 ATP → 2 NH_3_ + H_2_ + 16 ADP + 16 P_i_ (1)

**Figure 1:**
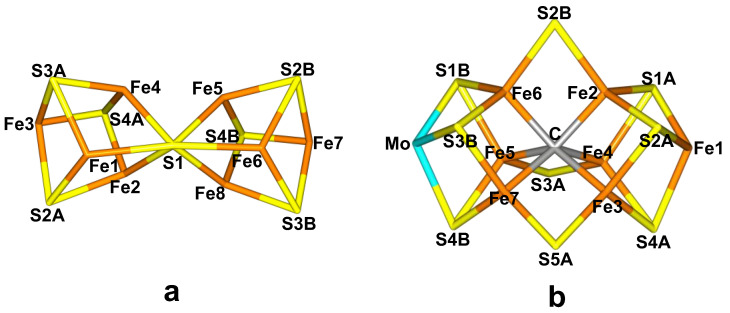
The cores of (**a**) the P-cluster (P^N^ state) and (**b**) the FeMo cluster (E_0_ state) with atom names indicated.

However, a few bacteria and archaea can perform the same conversion at ambient temperature and pressure. This is accomplished by the enzyme nitrogenase (EC 1.18/19.6.1) [2,4,5,6,7,8,9,10]. The reaction is still quite demanding, requiring 16 molecules of ATP, eight electrons and eight protons [2,4,5]: X-ray crystallographic studies have shown that nitrogenase contains two unusual iron–sulfur clusters [6,7,8,9,10]. The P-cluster, Fe_8_S_7_Cys_6_, is essentially two merged [4Fe–4S] clusters with a central sulfide ligand coordinating to six iron ions (Figure 1a). It is employed for electron transfer from the Fe-protein, which donates the electrons, to the active site. The latter is the FeMo cluster, which is a complicated MoFe_7_S_9_C(homocitrate) cluster, with a central carbide ion (Figure 1b) and connected to the protein by one cysteine and one histidine residue at the opposite ends of the trigonal prismatic cluster. There exist also alternative nitrogenases, in which the Mo ion is replaced by V or Fe [11].

Nitrogenase has been extensively studied by both experimental [2,3,4,5,12,13,14,15,16,17] and computational methods [18,19,20,21,22]. The reaction is normally described by the Lowe–Thorneley cycle [23], in which eight intermediates are recognised, E_0_–E_8_, based on the number of added electrons and protons. It has been shown that the resting E_0_ state needs to be reduced by three or four electrons before the N_2_ substrate can bind [2,3]. It is believed that the binding is facilitated by the reductive elimination of H_2_ from the cluster, explaining why H_2_ is a compulsory byproduct of the reaction. However, many details of the reaction are still unknown and many conflicting mechanisms have been suggested [3,18].

The Lowe–Thorneley cycle emphasizes the importance of electron and proton transfer in the reaction cycle of nitrogenase. The driving force of electron transfer is the redox potential. Unfortunately, it is hard to measure redox potentials of the FeMo cluster in nitrogenase because the reaction cannot be arrested at certain E*_n_* states [24]. The only certain redox potential is between the resting E_0_ state and a one-electron oxidised state, which is outside the Lowe–Thorneley cycle (we will denote it E_−1_), −0.042 V [5,24,25,26]. For the reduction of the resting state, a redox potential of −0.45 to −0.49 V have been reported, but they may represent a mixture of reduced states [24,25,27,28,29].

Redox potentials can also be calculated by computational methods. However, the accuracy is limited. For redox sites of the same type in variants of the same protein (i.e., from different organisms or mutations), calculations based on the Poisson–Boltzmann equation or similar methods may give mean errors of 0.03–0.11 V for relative redox potentials [30,31,32,33,34,35,36,37,38]. For absolute potentials and sites of different types, quantum mechanical (QM) calculations are needed and the accuracy is appreciably worse. Typical errors are 0.2–0.6 V [30,31,32,33,34] and a prediction of the potential of the FeMo cluster had an error of 1.3 V, leading to incorrect identification of the central carbide ion [39]. Even all-atom QM molecular dynamics and free-energy calculations did not give an accuracy better than 0.26 V [40].

Recently, we performed a comparison and calibration of various combined QM and molecular mechanics (QM/MM) methods to estimate redox potentials of 13 iron–sulfur clusters with 1–4 Fe ions [41]. We showed that the best results were obtained by QM-cluster calculations in a continuum solvent with a high dielectric constant, using a large QM model (~300 atoms), based on QM/MM structures. With such an approach, we obtained a mean absolute error of 0.17 V, after removal of a systematic error of 0.62 V. The maximum error among the 13 studied potentials was 0.44 V. However, even if the accuracy is rather mediocre, it is enough to make useful predictions, e.g., what redox couple is employed by the [4Fe–4S] ferredoxins.

In this study, we employ this calibrated approach to study redox potentials in nitrogenase. We study four issues: First, we examine whether the computational method works also for the more complicated P- and FeMo clusters in nitrogenase, with redox potentials that also involve proton transfer. Second, we obtain an independent check of the redox state and charge state of the FeMo cluster. Third, we examine the recent suggestion that the E_0_–E_4_ states should operate at a nearly constant potential, employing only a single redox couple [42,43]. Fourth, we study the redox potentials of different reaction mechanisms after the binding of the substrate (i.e., for the E_4_–E_8_ states).

## 2. Result and Discussion

### 2.1. Redox Potentials of the P-Cluster

We started the investigation with testing our methodology [41] on the P-cluster to see if the results are reliable also for the large iron–sulfur clusters in nitrogenase and for redox reactions involving protonation of the clusters. The P-cluster (Figure 1a) contains eight Fe ions and the resting P_N_ state has been shown to be the fully reduced ${\mathrm{Fe}}_{8}^{\mathrm{II}}$ state [44,45,46]. Three additional states have been experimentally observed, oxidised by 1–3 electrons [44,45,46]. They are denoted P^1+^–P^3+^. Only the first two states are believed to be involved in the catalytic mechanism, although there are some evidence that also the P^2+^ state may be used [46,47,48,49]. Crystallographic studies have shown that in the P^N^ state, the cluster is essentially two [4Fe–4S] clusters merged by the S1 sulfide ion that coordinates to six Fe ions (cf. Figure 1a). In the P^2+^ state, Ser-188D becomes deprotonated and coordinates to Fe6 [48,50,51]. Likewise, the backbone N atom of Cys-88 (the sidechain of which is one of the ligands to the P-cluster) also becomes deprotonated and coordinates to Fe5 [48,50,51]. This leads to cleavage of the Fe5–S1 and Fe6–S1 bonds. The same structure is expected for the P^3+^ state. The structure of the P^1+^ state is more uncertain, because redox titrations indicated that only the P^2+^/P^1+^ redox-couple is pH-dependent, whereas no evidence was found for a coupled electron- and proton-transfer for the P^1+^/P^N^ couple [52,53]. However, a recent crystal structure was interpreted to contain deprotonated Ser-188D and protonated Cys-88, although it is probably a mixture of the P^1+^ and the P^2+^ states [54,55,56].

Six states of the P-cluster were considered in this study as are described in Table 1. Each state was first QM/MM optimised with the small QM system and then subjected to a single-point energy calculation with the large QM system in a COSMO continuum solvent with a dielectric constant of 80. The QM/MM structures and the best BS states were taken from our previous publication [55]. The calculated redox potentials are listed in Table 2 and they are compared to experimentally measured redox potentials [5] in order to gauge the accuracy of the method when applied to the P-clusters of the nitrogenase. For the P^1+^ state, we tested three different protonation states.

It can be seen that the redox couples P^2+^ → P^3+^ and P^N^H_2_ → P^1+^H give errors of 0.05 and 0.33 V, respectively, compared to experimental potentials. These are within the range of errors observed in our previous study (maximum error 0.44 V) [41]. This is quite satisfying, especially considering that one of the considered redox potentials for the P-cluster involves coupled redox and protonation reaction, whereas the calibration study involved only pure redox reactions. This gives us confidence to apply the method also to the FeMo cluster.

For the P^1+^/P^N^ redox potential, the calculated result is essentially independent on whether we include the proton transfer or not in the calculations (the P^N^H_2_ → P^1+^H_2_ and P^N^H_2_ → P^1+^H give calculated redox potentials that differ by only 0.02 V). This is in agreement with the experimental observation that the P^1+^/P^N^ redox potential is pH-independent [52,53] and may solve the enigma why redox titrations did not observe any pH-dependence although crystal structures indicate that a deprotonation should be involved in this redox reaction [54,55,56].

For the P^1+^H → P^2+^ transition, the comparison with experimental results is somewhat harder, because the measured redox potential changes with pH, from −0.224 V at pH 6.0 to −0.348 V at pH 8.5 [52]. This has been interpreted to reflect the deprotonation of the backbone amide group of Cys-88: At very low pH, it is protonated in both the P^1+^ and P^2+^ states, whereas at high pH, it is deprotonated in both redox states. At intermediate pH (i.e., in the measured range), it is protonated in the P^1+^ state and deprotonated in the P^2+^ state [52]. Our calculations confirm these suggestions: We obtain a more positive potential for the P^2+^/P^1+^H couple ($E_{\mathrm{corr}}^{o}$ = 0.1 V) than for the P^2+^/P^1+^ couple ($E_{\mathrm{corr}}^{o}$ = −1.1 V). However, the restricted pH range of the measured potentials makes it hard to make a more detailed judgement of the calculated potentials. The experiments indicate that the redox potential of the pure P^1+^H → P^2+^ transition is larger than −0.224 V, indicating an error of less than 0.36 V for our calculated potential. Likewise, the redox potential of the pure P^1+^ → P^2+^ transition is more negative than −0.348 V, indicating an error of less than 0.72 V. Thus, our calculations confirm the experimental observation that the P^1+^ → P^2+^ transition involves a proton transfer [52,53], but we cannot obtain any detailed estimate of the error for this redox couple.

### 2.2. Accuracy of the Redox Potential of the FeMo Cluster and Oxidation Level of the Cluster

As mentioned above, only two experimental redox potentials have been reported for the FeMo cluster [24,25]. A potential of −0.042 V has been measured between the resting state and a one-electron oxidised state [26,45] (not involved in the normal reaction mechanism). Redox potentials for more reduced states are harder to measure, because reduction starts substrate or proton reduction. No firm measurement is available, but the potential between the resting state and a steady-state reduced state (which my represent more than one reduced state) has been estimated in four studies between −0.45 and −0.49 V [24,25,27,28,29]. In two cases, another potential of −0.30 to −0.32 V was also reported, but it may come from the P-cluster [24].

For the FeMo cluster, we wanted to study two related questions, viz. whether we can reproduce these potentials with our QM calculations, within the accuracy of the method, and whether we can identify the correct redox couple of the FeMo cluster. Recent Mössbauer, anomalous dispersion and QM investigations have suggested that the resting E_0_ state of the FeMo cluster is in the ${\mathrm{Mo}}^{\mathrm{III}}{\mathrm{Fe}}_{3}^{\mathrm{II}}{\mathrm{Fe}}_{4}^{\mathrm{III}}$ oxidation state [13,42,57,58]. However, this gives a large net negative charge of the cluster and its direct ligands (−5 *e*), which is only partly neutralised by two nearby arginine residues. Moreover, protonation energies of various groups of the FeMo cluster are unexpectedly large [59,60]. Therefore, an independent confirmation of the oxidation-state assignment is desirable.

To this end, twelve states of the FeMo cluster were considered, described in Table 3. We studied the resting E_0_ state, together with the one-electron oxidised and one-electron reduced states. For the latter, we considered both a structure with no protons added and a state with a proton added on the S2B µ_2_ bridging sulfide ion, as has been suggested by several QM investigations [18,59] and is also supported by experimental studies [61,62]. For the standard oxidation-state assignment (${\mathrm{Mo}}^{\mathrm{III}}{\mathrm{Fe}}_{3}^{\mathrm{II}}{\mathrm{Fe}}_{4}^{\mathrm{III}}$ for E_0_), these states are denoted E_0_, E_−1_, E_1_ and E_1_H. In addition, we considered two alternative charge states, either with two more or two electrons less (experimentally the resting state is a quartet [2,3], i.e., with an odd number of electrons, so electrons need to be added or removed in pairs). These states are called A_0_, A_−1_, A_1_ and A_1_H when two electrons were added (i.e., giving ${\mathrm{Mo}}^{\mathrm{III}}{\mathrm{Fe}}_{5}^{\mathrm{II}}{\mathrm{Fe}}_{2}^{\mathrm{III}}$ for the A_0_ state) and R_0_, R_−1_, R_1_ and R_1_H when two electrons were removed (i.e., giving ${\mathrm{Mo}}^{\mathrm{III}}{\mathrm{Fe}}_{1}^{\mathrm{II}}{\mathrm{Fe}}_{6}^{\mathrm{III}}$ for the R_0_ state; note that R_1_ = E_−1_ and A_−1_ = E_1_). The calculated redox potentials are listed in Table 4.

It can be seen that with the standard charge state (${\mathrm{Mo}}^{\mathrm{III}}{\mathrm{Fe}}_{3}^{\mathrm{II}}{\mathrm{Fe}}_{4}^{\mathrm{III}}$ for E_0_), our calculations reproduce the two experimental redox potentials with errors of 0.17 and 0.19 V, i.e., well within the error range observed in our previous study (maximum error 0.44 V) [41] and also for the P-cluster. However, the good results are observed only if it is assumed that the reduction of E_0_ is accompanied by the uptake of by a proton (i.e., E_0_ → E_1_H; for E_0_ → E_1_, the error is 0.6 V), showing that the calculations confirm that a proton transfer is involved in the redox reaction.

If we instead consider a FeMo cluster with two electrons less (i.e., with a ${\mathrm{Mo}}^{\mathrm{III}}{\mathrm{Fe}}_{1}^{\mathrm{II}}{\mathrm{Fe}}_{6}^{\mathrm{III}}$ assignment for the resting state, here called R_0_), the calculated potentials reproduce the experimental redox potentials worse: The R_−1_ → R_0_ transition gives and error of 2.0 V, much larger than the maximum error in our previous study [41], whereas R_0_ → R_1_H gives an error of 0.43 V.

Likewise, if we instead add two extra electrons (${\mathrm{Mo}}^{\mathrm{III}}{\mathrm{Fe}}_{5}^{\mathrm{II}}{\mathrm{Fe}}_{2}^{\mathrm{III}}$ for the resting state), we get a very large error (over 6 V) for the A_−1_ → A_0_ potential. For the A_0_ → A_1_H transition, the error is smaller, −0.48 V, but it is still somewhat larger than the maximum error observed in our calibration study. Thus, our calculations confirm that ${\mathrm{Mo}}^{\mathrm{III}}{\mathrm{Fe}}_{3}^{\mathrm{II}}{\mathrm{Fe}}_{4}^{\mathrm{III}}$ is the proper redox assignment for E_0_. Apparently, the E_−1_/E_0_ redox potential is more sensitive to the involved redox couple than the E_0_/E_1_H potential. The calculations also confirm that the E_1_ state is protonated.

### 2.3. Redox Potentials of the E_0_–E_4_ States of the FeMo Cluster

Next, we studied also the E_0_–E_4_ states of the FeMo cluster from Lowe–Thorneley reaction cycle. The aim was to examine the suggestion that all the E_0_–E_4_ states should have similar redox potentials, because they use only two formal redox states, ${\mathrm{Mo}}^{\mathrm{III}}{\mathrm{Fe}}_{3}^{\mathrm{II}}{\mathrm{Fe}}_{4}^{\mathrm{III}}$ and ${\mathrm{Mo}}^{\mathrm{III}}{\mathrm{Fe}}_{4}^{\mathrm{II}}{\mathrm{Fe}}_{3}^{\mathrm{III}}$ [42,43]. This is suggested to be accomplished by the added protons, which bind to Fe ions in the E_2_ and E_4_ states, thereby formally becoming hydride ions and changing the oxidation state of the Fe ions by two. Thus, the five states E_0_–E_4_ would formally be ${\mathrm{Fe}}_{3}^{\mathrm{II}}{\mathrm{Fe}}_{4}^{\mathrm{III}}$, ${\mathrm{Fe}}_{4}^{\mathrm{II}}{\mathrm{Fe}}_{3}^{\mathrm{III}}H^{+}$, ${\mathrm{Fe}}_{3}^{\mathrm{II}}{\mathrm{Fe}}_{4}^{\mathrm{III}}H^{+}H^{-}$, ${\mathrm{Fe}}_{4}^{\mathrm{II}}{\mathrm{Fe}}_{3}^{\mathrm{III}}H_{2}^{+}H^{-}$ and ${\mathrm{Fe}}_{3}^{\mathrm{II}}{\mathrm{Fe}}_{4}^{\mathrm{III}}H_{2}^{+}H_{2}^{-}$ (leaving out Mo, which always is in the +III state).

We used mainly structures from previous studies [59,63,64,65,66,67] and included a few alternative structures for each state (except for E_0_ and E_1_H, for which there is a reasonable consensus), to see if we can discriminate between different possibilities using the redox potentials. The various structures are described in Table 5 (they are also shown in Figure S3) and the calculated redox potentials are listed in Table 6. We use the redox potential of the E_0_/E_1_H couple as a reference ($\Delta E_{\mathrm{calc}}^{o}=E_{\mathrm{calc}}^{o}-\Delta E_{\mathrm{calc}}^{o}\left(E_{0}/E_{1}H\right))$ to judge if all transitions have similar redox potentials.

In our previous studies, we found that with the TPSS functional, the best E_2_H_2_ structure has a proton on S2B and a hydride ion bridging Fe2 and Fe6 [59,64]. It can be seen that the estimated redox potential for the E_1_H → E_2_H_2_ transition is similar to that of the E_0_ → E_1_H transition, only 0.08 V less negative. This confirms the conjecture that the FeMo cluster in the early E*_n_* states should have a constant redox potential.

If we instead use another structure for the E_2_H_2_ state with the extra two protons bound to S2B and as a hydride ion bridging Fe2 and Fe6, but with S2B dissociated from Fe2 (but not from Fe6; called E_2_H_2_′), the redox potential for the E_1_H → E_2_H_2_′ transition decreases by 0.25 V. This simply reflects that with TPSS-D4/def2-SV(P) and the 399-atom redox model, the E_2_H_2_′ structure is 0.25 eV (24 kJ/mol) less stable than the E_2_H_2_ structure. For the 184-atom QM/MM model, the difference is 15 kJ/mol. In our previous study, we showed that the relative stability of the structures with the protonated S2B group bridging Fe2 and Fe6 or dissociated from one of the two iron ions depends on what DFT functional is used [64]. For example, with the TPSSh functional, the E_2_H_2_′ structure is instead 11 kJ/mol more stable. Consequently, the redox potentials will also depend on the QM method used, with differences of ~0.3 V.

If we instead use a structure for the E_2_H_2_ state with the two protons on S2B and S5A (E_2_H_2_″), the calculated redox potential is 0.08 V more negative than with the most stable E_2_H_2_ state. Again, this reflects that this protonation state is 9 kJ/mol less stable. However, it also shows that the formal oxidation states in the FeMo cluster have only a minor influence on the redox potential. The E_2_H_2_″ state involves two protons on sulfide groups and therefore represents a doubly reduced formal ${\mathrm{Fe}}_{5}^{\mathrm{II}}{\mathrm{Fe}}_{2}^{\mathrm{III}}H_{2}^{+}$ state in contrast to the ${\mathrm{Fe}}_{3}^{\mathrm{II}}{\mathrm{Fe}}_{4}^{\mathrm{III}}H^{+}H^{-}$ state for E_2_H_2_ and E_2_H_2_′. This shows that as long as the various protonation states have similar relative energies, they will also have similar redox potentials, showing that there is no major difference between different formal oxidation states of the Fe ions. This is in line with suggestions by Dance, who has pointed out that it is misleading to make a sharp contrast between protons on sulfides and hydride ions on Fe ions, because there is only a small difference in the charge populations on the H atom [18].

For the E_3_H_3_ state, we used a structure with the third proton bridging Fe3 and Fe7 (in addition a proton on S2B and a hydride ion bridging Fe2 and Fe6). The estimated redox potential for the E_2_H_2_ → E_3_H_3_ transition is 0.19 V more negative than that of the E_0_ → E_1_H transition. This is well within the maximum error in our calibration study 0.44 V [41], and therefore still in agreement with the expectation that the electrons can be donated to the FeMo cluster at a constant redox potential. We tested also another structure, taken from our previous systematic study [59], in which the third proton bound to Fe5 instead (E_3_H_3_′). It gave a 0.35 V more negative redox potential (i.e., further away from that of the E_0_ → E_1_H transition), reflecting that this structure is less stable. Interestingly, we found in contrast to our previous study that the broken-symmetry BS-14 state was more favourable for both these structures with the large QM model used in the redox calculations (but only for the best structure with the smaller QM/MM-optimised model).

For the E_3_H_3_ → E_4_H_4_ transition, our estimated redox potential is 0.41 V more positive than that of the E_0_ → E_1_H transition. This is within the maximum error in our calibration study [41], but considering that the redox potential of the E_2_H_2_/E_3_H_3_ couple was a bit to negative and this redox potential is a bit too positive, it might indicate that we have not yet found the best structure for the E_3_ state. For E_4_H_4_, we employed the best structure in our previous investigation of this state [63], viz. a structure with two protons on S2B and S5A and two hydride ions bridging Fe2/6 and Fe3/7. There are several possible conformations of such a structure. The best one has all H atoms pointing towards S3A, except the Fe3/7 hydride, which points towards S2B (all structures are shown in Figure S3). If we instead use a structure with the Fe2/6 hydride on the other side of S2B, i.e., the same face as the Fe2/6 hydride, the redox potential becomes 0.23 V more negative, reflecting that such a structure is 22 kJ/mol less stable. If we instead use the structure suggested by Hoffman and coworkers [2,21], i.e., with all four H atoms on the same face of the cluster (i.e., the two protons on S2B and S5A pointing in the opposite direction compared to E_4_H_4_ and E_4_H_4_′ structures), the redox potential becomes even more negative by 0.24 V. Likewise, if we use the best structure in our first investigation of the E_4_H_4_ state [59] (with H atoms on S2B, Fe2/6, Fe5 and Fe6), the redox potential becomes 0.15 V even more negative, reflecting that this structure is 59 kJ/mol less stable than the best state.

In conclusion, we find that for all four calculated redox potentials for the E_0_–E_4_ states are similar, within 0.41 V, i.e., within the accuracy of our method. This confirm the expectation that the redox potentials should be similar [42,43] so that they can accept electrons from the same source. However, the results are very sensitive to which structures are employed and which QM method and broken-symmetry state is used. On the other hand, we show that the formal oxidation states of the Fe ions (the number of protons on sulfide ions or hydride ions on Fe) is less important for the redox potentials, contrary to the suggestion that the redox potential depends on the formal oxidation state of the Fe ions [43,68].

### 2.4. Redox Potentials of the E_4_–E_8_ States of the FeMo Cluster

Finally, we studied also redox potentials of the FeMo cluster in the later part of the reaction, after binding of N_2_ and its protonation to N_2_H_2_. In previous studies, we have suggested thermodynamically stable structures for the E_4_N_2_H_2_ to E_8_NH_3_ states, either with S2B bound or dissociated from the cluster [65,66,67]. We use these structures also in this study. They are described in Table 5 and are shown in Figure S3. In Table 6, the calculated redox potentials for both scenarios are presented.

It can be seen that both with and without S2B, the calculated redox potentials are all less negative than that for the E_0_/E_1_H couple, by 0.2–2.65 V. This reflects that once N_2_H_2_ has been formed, the following reactions are quite facile. Electrons tend to move towards sites with a more positive redox potential. Therefore, redox potentials that are more positive than those of the E_0_/E_1_H couple indicate that the electron transfer is more exothermic than in the E_0_ → E_1_ step. Thus, the electron transfers of the E_4_–E_8_ steps of the nitrogenase reaction are more downhill than those of the E_0_–E_4_ reactions. However, it might also indicate that the assumption that the bound N_2_ directly is protonated may be incorrect. In fact, the first protonation of N_2_ is the hardest step in the reduction of N_2_ to ammonia [69], and it is possible that it requires further reduction of the FeMo cluster before it is feasible (this part of the reaction was not studied in our previous studies). Our results indicate that this should be further studied.

The relative sizes of the four redox potentials for the E_4_–E_8_ states are also rather independent on whether S2B remains bound or is dissociated: The potentials of the first and third steps E_4_ → E_5_ and E_6_ → E_7_ are most positive, especially the latter, whereas the redox potentials of the other two steps are closer to that of the E_0_/E_1_H couple. For the four reductions with S2B still bound, our calculated redox potentials are 1.13, 0.41, 2.02 and 0.57 V, whereas with S2B dissociated, the four calculated redox potentials are 0.97, 0.20, 2.65 and 0.19 V (i.e., with a somewhat larger variation). The similarity of the trends for the two sets of potentials is conspicuous considering that the N–N bond is cleaved in the E_5_N_2_H_3_ → E_6_N_2_H_4_ transition with S2B bound, but in the E_6_N_2_H_4_ → E_7_N_2_H_5_ transition when S2B has dissociated.

In conclusion, our results show that later part of the reaction mechanism of Mo-nitrogenase give redox reactions that are more exothermic than that of the E_0_/E_1_H redox couple. Thus, we see no evidence that a stronger driving force is needed for the reaction, as has been suggested by Siegbahn [22]. Moreover, there is no large difference between the mechanisms with S2B bound or dissociated.

## 3. Methods

### 3.1. The Protein

The calculations were based on the 1.0-Å crystal structure of Mo nitrogenase from *Azotobacter vinelandii* (PDB code 3U7Q) [8]. The setup of the protein is identical to that of our previous studies [60,63,69,70]. The entire heterotetramer was considered in the calculations and the quantum mechanical (QM) calculations were concentrated on the FeMo clusters in the C subunit because there is a buried imidazole molecule from the solvent rather close to the active site (~11 Å) in the A subunit. The metal clusters not involved in the QM calculations were modelled by MM in the fully reduced and E_0_ resting states, respectively, using a QM charge model [70]. The protonation states of all residues were the same as before [70], and the homocitrate ligand was modelled in the singly protonated state with a proton shared between the hydroxyl group (O7 that coordinates to Mo) and the O1 carboxylate atom [57,70]. The protein was solvated in a sphere with a radius of 65 Å around the geometrical centre of the protein. Cl^−^ and Na^+^ ions were added to an ionic strength of 0.2 M [71]. The final system contained 133 915 atoms. For the protein, we used the Amber ff14SB force field [72] and water molecules were described by the TIP3P model [73]. The metal sites [70,74] were treated by a non-bonded model [75] and charges were obtained with the restrained electrostatic potential method [76].

### 3.2. QM Calculations

All QM calculations were performed with the Turbomole software (version 7.5) [77]. All calculations were performed with the TPSS [78] functional with the def2-SV(P) basis set [79], a combination that gave the best relative redox potentials in our previous study (lowest mean absolute deviation and maximum error, after removal of the mean signed error; B3LYP or larger basis sets gave more than twice as large mean absolute deviations and maximum errors, but a slightly smaller mean signed error) [41]. The calculations were sped up by expanding the Coulomb interactions in an auxiliary basis set, the resolution-of-identity (RI) approximation [80,81]. Empirical dispersion corrections were included with the DFT-D4 approach [82], as implemented in Turbomole. QM calculations were performed on both the FeMo cluster and the P-cluster, and two different sizes of the QM systems were employed, one smaller for QM/MM geometry optimisations and one larger for the redox-potential calculations.

In the QM/MM geometry optimisations, the P-cluster was modelled as Fe_8_S_7_Cys_6_, with the five of the Cys ligands modelled by CH_3_S^−^_,_ whereas Cys–88 was modelled by CH_3_CONHCH_2_CH_2_S^−^, because the backbone amide group is deprotonated and coordinates to Fe5 in some the more oxidised states. Likewise, Ser-188D (i.e., belonging to subunit D, rather than C for all the other numbered residues) was included in the model as CH_3_OH because it is deprototated and coordinates to Fe6 in the oxidised states. The model contained 64 atoms for the fully protonated state and it is shown in Figure S1a in the Supporting Information. All QM/MM structures of the P-cluster were taken from our previous study [55].

The FeMo cluster was modelled by MoFe_7_S_9_C(homocitrate)(CH_3_S) (imidazole) in the QM/MM calculations, where the two last groups are models of Cys-275 and His-442. In addition, all groups that form hydrogen bonds to the FeMo cluster were also included in the QM model, viz. Arg-96, Gln-191 and His-195 (sidechains), Ser-278 and Arg-359 (both backbone and sidechain, including the CA and C and O atoms from Arg-277), Gly-356, Gly-357 and Leu-358 (backbone, including the CA and C and O atoms from Ile-355), as well as two water molecules. Finally, the sidechains of Glu-380, Val-70 and Phe-381 were also included. This QM system involved 191 atoms and is shown in Figure S1b.

For the redox-potential calculations, we used the largest QM system suggested in our previous investigation [41]. It included all functional groups in the proteins with any atom within 3.5 Å of a minimal QM system, consisting of all metal and sulfide ions together with all direct ligands (Cys, His and homocitrate). These QM systems were set up using our local program for BigQM calculations (changepdb) [83]. They contained ~400 atoms for the FeMo cluster and ~500 atoms for the P-cluster, and are shown in Figure S2. The calculations were based on the QM/MM-optimised structures.

In the redox calculations, the QM system was immersed into a continuum solvent, employing the conductor-like screening model (COSMO) [84,85] implemented in Turbomole. The default optimised COSMO atomic radii and a water solvent radius of 1.3 Å were employed to construct the solvent-accessible surface cavity [86], whereas a radius of 2.0 Å was used for Fe and Mo [87]. Structures for the QM + COSMO calculations were taken directly from the QM/MM calculations without further optimisation. The dielectric constant was 80, which gave the best redox potentials in our previous study [41].

Redox potentials (*E*º) were calculated according to

*E*º = *E*(ox) − *E*(red) − *c* (2)

where *E*(ox) and *E*(red) are the energies of the oxidised and reduced states, and *c* is a correction factor (4.28 eV) to place the potentials on the scale of the standard hydrogen electrode [88]. The actual value of this factor has been much discussed and values between 4.05–4.44 eV have been suggested [88,89]. To avoid this problem, we use the method calibrated in our previous study to 13 different iron–sulfur clusters [41]. Therefore, we subtract from the *c* constant the mean signed error (MSE) obtained in this calibration study, MSE = −0.62 V or we subtract the calculated potential of the E_0_ → E_1_ transition from the redox potentials of the other states.

In some cases, it is known or assumed that the cluster takes up a proton during or after the electron transfer. In those cases, it was assumed that the proton comes from an imidazole molecule, studied with the same QM method in a COSMO continuum solvation solvent with the dielectric constant of water (80). Thus, we assume that the proton comes from a group with a pK_a_ close to 7 (6.95). This changes the calculated redox potentials by −12.47 V.

The electronic structure of all QM systems was obtained with the broken-symmetry (BS) approach [90]: Each of the seven or eight Fe ions was modelled in the high-spin state, with either a surplus of α or β spin. Such a state can be selected in many different ways, giving rise to different BS states, which are specified by giving the number of the Fe ions with minority spin (the numbering of the Fe ions is shown in Figure 1) [55,60]. The various BS states were obtained either by swapping the coordinates of the Fe ions [91] or with the fragment approach by Szilagyi and Winslow [92].

### 3.3. QM/MM Calculations

QM/MM calculations were performed with the ComQum software [93,94]. In this approach, the protein and solvent are split into two subsystems: System 1 (the QM region) was relaxed by QM methods. System 2 contained the remaining part of the protein and the solvent, and it was kept fixed at the original coordinates (equilibrated crystal structure [70], to avoid the risk that different calculations end up in different local minima).

In the QM calculations, system 1 was represented by a wavefunction, whereas all the other atoms were represented by an array of partial point charges, one for each atom, taken from the MM setup. Thereby, the polarisation of the QM system by the surroundings is included in a self-consistent manner (electrostatic embedding). When there is a bond between systems 1 and 2 (a junction), the hydrogen link-atom approach was employed: The QM system was capped with hydrogen atoms, the positions of which are linearly related to the corresponding carbon atoms (carbon link atoms, CL) in the full system [93,95]. All atoms were included in the point-charge model, except the CL atoms [96]. ComQum employs a subtractive scheme with van der Waals link-atom corrections [97]. No cut-off is used for the QM and QM–MM interactions. The geometry optimisations were continued until the energy change between two iterations was less than 2.6 J/mol (10^−6^ a.u.) and the maximum norm of the Cartesian gradients was below 10^−3^ a.u.

## 4. Conclusions

In this study, we have investigated what information we can get from calculated redox potentials of the two metal clusters in Mo-nitrogenase. We employ our calibrated approach to calculate redox potentials for iron–sulfur clusters involving QM-cluster calculations in a continuum solvent with large QM models (400–500 atoms), based on structures from QM/MM optimisations [41]. We obtain several interesting results:

The calculations on the P-cluster show that our method gives approximately the same accuracy as for the simple iron–sulfur clusters with 1–4 Fe ions, with a maximum error of 0.33 V (0.44 V in our previous study [41]). This shows that the calculations are accurate also for the larger P-cluster and for redox reactions that include proton transfers.

The calculations confirm that the P^1+^ → P^2+^ transition involves a proton transfer (i.e., P^1+^H → P^2+^), as is also suggested by electrochemical and crystallographic studies [8,48,50,51,54,55,56].

The calculations show that the P^1+^H_2_/P^N^H_2_ and P^1+^H/P^N^H_2_ redox couples give very similar redox potentials, which may explain the experimental enigma that redox titrations do not show any pH dependence of the P^1+^/P^N^ redox potential [52,53], although crystal structures indicate that also the P^N^ → P^1+^ transition should involve a proton transfer [54,55,56].

For the FeMo-cluster, the calculations unambiguously identify ${\mathrm{Mo}}^{\mathrm{III}}{\mathrm{Fe}}_{3}^{\mathrm{II}}{\mathrm{Fe}}_{4}^{\mathrm{III}}$ as the proper formal oxidation state for the resting E_0_ state of the protein. This provides an independent confirmation of this oxidation state, also suggested by previous experimental and QM studies [13,42,57,58].

The calculations agree with experiments only if it is assumed that a proton is taken up together with the electron in the E_0_ → E_1_ reaction.

The calculations confirm that the E_0_ to E_4_H_4_ states all have similar redox potentials (within 0.41 V, i.e., lower than the estimated maximum error of the method), as expected for a site that should receive electrons from the same donor.

However, there is no major difference in the redox potentials between structures with protons on the µ_2_-bridging sulfide ions or hydride ions on the Fe ions (for E_2_H_2_ the difference is only 0.08 V). This shows that there is only minor differences between hydrogen atoms bound to S or Fe ions, as previously has been advocated by Dance [18] and that the formal oxidation states of the Fe ions are no good indicators of the redox potentials.

The redox potentials of the later steps of the reaction mechanism (E_4_N_2_H_2_ to E_8_) are more positive than that of the resting state (E_0_/E_1_H), showing that the reactions are more exothermic. The trends in the potentials do not change if S2B remains bound to the cluster or if it dissociates. This shows that there is no need of a significantly more negative potential for the Fe protein than measured.

The calculated redox potentials strongly depend on the structures used (the positions of the added protons), the broken-symmetry states and the QM method employed.

Thus, our calculations show that quite strong predictions can be provided by redox-potential calculations, even if the accuracy is rather poor compared to experimental measurements (a maximum error of 0.44 V), provided that the calculations are calibrated so that the expected errors are known. Such calculations can also identify possible problems in suggested reaction mechanisms. Our calculations also allow us to identify what redox reactions involve coupled electron and proton transfer, which is crucial to identify the detailed reaction mechanism of nitrogenase. Moreover, we have been able to explain the enigma why the P^1+^/P^N^ redox potential is not pH dependent [52,53], although the crystal structures show that the a deprotonation reaction is involved [54,55,56].

## Figures and Tables

**Table 1:** The various states studied for the P-cluster. The table lists an abbreviation (Abb.) for each state, the protonation status for Cys-88 and Ser-188D (P = protonated, D = deprotonated), the spin state (*S*), the BS state (specifying Fe ions with minority spin) and the net charge of the QM systems (ch; same for both for QM/MM and for the redox calculations).

State	Abb.	Cys-88	Ser-188	*S*	BS	ch
reduced	P^N^H_2_	P	P	0	1247	−4
one-electron oxidised	P^1+^H_2_	P	P	½	1247	−3
	P^1+^H	P	D	½	1247	−4
	P^1+^	D	D	½	1247	−5
two-electron oxidised	P^2+^	D	D	4	358	−4
three-electron oxidised	P^3+^	D	D	7/2	358	−3

**Table 2:** Redox potentials of five redox couples for the P-cluster in V. $E_{\mathrm{calc}}^{o}$ is the raw redox potentials from the COSMO calculations, $E_{\mathrm{corr}}^{o}$ are calculated redox potentials corrected by the mean signed error (MSE = −0.62 V) from our previous study [41] (i.e., $E_{\mathrm{corr}}^{o}=E_{\mathrm{calc}}^{o}+0.62$ V), $E_{\exp}^{o}$ is the experimental redox potentials [5,24,25,45] and $E_{\mathrm{err}}^{o}$ is the error for the various calculations. Results with $E_{\mathrm{err}}^{o}$ < 0.44 V are marked in bold face.

Redox Couple	$\;\;\;E_{\mathrm{calc}}^{o}$	$E_{\mathrm{corr}}^{o}$	$E_{\exp}^{o}$	$\;E_{\mathrm{err}}^{o}$
P^N^H_2_ → P^1+^H_2_	−0.62	0.00	−0.309	**0.31**
P^N^H_2_ → P^1+^H	−0.60	0.02	−0.309	**0.33**
P^1+^H → P^2+^	−0.48	0.14	<−0.224 ^a^	**<0.36**
P^1+^ → P^2+^	−1.69	−1.07	<−0.348 ^a^	<0.72
P^2+^ → P^3+^	−0.58	0.04	0.09	**−0.05**

^a^ The measured redox potential for the P^2+^/P^1+^ couple is pH dependent, decreasing from −0.224 V at pH 6.0 to −0.348 V at pH 8.5 (cf. the text) [52].

**Table 3:** States studied for the resting, one-electron reduced or one-electron oxidised states of the FeMo cluster. The table lists the protonation status of the S2B atom (P = protonated, D = deprotonated), the spin state (*S*), the BS state (specifying Fe ions with minority spin), and the net charge of the QM systems (ch; same for both for QM/MM and for the redox calculations).

State	S2B	*S*	BS	ch
R_−1_	D	1	235	0
R_0_	D	3/2	235	−1
R_1_	D	2	235	−2
R_1_H	P	2	235	−1
E_−1_	D	1	235	−2
E_0_	D	3/2	235	−3
E_1_	D	2	235	−4
E_1_H	P	2	235	−3
A_−1_	D	2	235	−4
A_0_	D	3/2	235	−5
A_1_	D	2	235	−6
A_1_H	P	2	235	−5

**Table 4:** Redox potentials for various redox couples of the FeMo cluster in V. $E_{\mathrm{calc}}^{o}$ is the raw redox potentials, $E_{\mathrm{corr}}^{o}=E_{\mathrm{calc}}^{o}+0.62$ V (the MSE in our previous study [41]), $E_{\exp}^{o}$ is the experimental redox potentials (for the E_0_/E_1_ couple, we used −0.47 V, which is in the middle of the range of reported values) [5,24,25,27,28,29] and $E_{\mathrm{err}}^{o}$ is the error for the various calculations. Results with $\;E_{\mathrm{err}}^{o}$ < 0.44 V are marked in bold face.

Transition	$E_{\mathrm{calc}}^{o}$	$E_{\mathrm{corr}}^{o}$	$E_{\exp}^{o}$	$\;E_{\mathrm{err}}^{o}$
R_−1_ → R_0_	1.31	1.93	−0.042	1.98
R_0_ → R_1_	0.42	1.04	(−0.47)	1.51
R_0_ → R_1_H	−0.66	−0.04	−0.47	**0.43**
E_−1_ → E_0_	−0.49	0.13	−0.042	**0.17**
E_0_ → E_1_	−1.69	−1.07	(−0.47)	−0.60
E_0_ → E_1_H	−1.28	−0.66	−0.47	**−0.19**
A_−1_ → A_0_	−6.88	−6.29	−0.042	−6.21
A_0_ → A_1_	−3.46	−2.84	(−0.47)	−2.37
A_0_ → A_1_H	−1.57	−0.95	−0.47	−0.48

**Table 5:** Structures studied for the E_0_–E_8_ states of the FeMo cluster. The table lists the protonated atoms or the added substrate, the spin state (*S*), the BS state (specifying the Fe ions with minority spin), and the net charge of the QM systems (ch; same for both for QM/MM and for the redox calculations).

State	Protonated Atoms/Substrate	*S*	BS	ch
E_0_	–	3/2	235	−3
E_1_H	S2B(3)	2	235	−3
E_2_H_2_	S2B(3), Fe2/6(5)	3/2	247	−3
E_2_H_2_′	S2B (dissoc from Fe2), Fe2/6(5)	3/2	247	−3
E_2_H_2_″	S2B(3), S5A(3)	3/2	247	−3
E_3_H_3_	S2B(3), Fe2/6(5), Fe3/7(2)	1	14	−3
E_3_H_3_′	S2B(3), Fe2/6(5), Fe5	1	14	−3
E_4_H_4_	S2B(3), Fe2/6(3), Fe3/7(2), S5A(3)	½	14	−3
E_4_H_4_′	S2B(3), Fe2/6(5), Fe3/7(2), S5A(3)	½	14	−3
E_4_H_4_″	S2B(5), Fe2/6(5), Fe3/7(2), S5A(2)	½	14	−3
E_4_H_4_″′	S2B(3), Fe2/6(3), Fe6, Fe5	½	14	−3
With S2B still bound				
E_4_N_2_H_2_	HNNH_2_ (proton from HCA)	½	147	−3
E_5_N_2_H_3_	H_2_NNH_2_ (proton from HCA)	1	147	−3
E_6_N_2_H_4_	NH_2_ + NH_3_ (proton from HCA)	½	147	−3
E_6_NH	NH_2_ (proton from HCA)	½	147	−3
E_7_NH_2_	NH_3_ (proton from HCA)	1	147	−3
E_8_NH_3_	NH_3_	½	147	−3
With S2B dissociated				
E_4_N_2_H_2_′	NNH_2_	½	147	−1
E_5_N_2_H_3_′	HNNH_2_	1	147	−1
E_6_N_2_H_4_′	H_2_NNH_2_	½	147	−1
E_7_N_2_H_5_′	NH_2_ + NH_3_	0	147	−1
E_7_NH_2_′	NH_2_	1	147	−1
E_8_NH_3_′	NH_3_	½	147	−1

**Table 6:** Calculated redox potentials for the E_0_–E_8_ states of the FeMo cluster. The last column ($\Delta E_{\mathrm{calc}}^{o}$ ) reports the difference in the calculated redox potential compared to that of the E_0_ → E_1_H transition. Redox potentials for the most favourable structures of the first four transitions are shown in bold face.

Transition	$E_{\mathrm{calc}}^{o}$	$\Delta E_{\mathrm{calc}}^{o}$
E_0_ → E_1_H	**−1.28**	**0.00**
E_1_H → E_2_H_2_	**−1.20**	**0.08**
E_1_H → E_2_H_2_′	−1.45	−0.17
E_1_H → E_2_H_2_″	−1.29	0.00
E_2_H_2_ → E_3_H_3_	**−1.47**	**−0.19**
E_2_H_2_ → E_3_H_3_′	−1.81	−0.53
E_3_H_3_ → E_4_H_4_	**−0.87**	**0.41**
E_3_H_3_ → E_4_H_4_′	−1.10	0.18
E_3_H_3_ → E_4_H_4_″	−1.34	−0.06
E_3_H_3_ → E_4_H_4_″′	−1.49	−0.21
With S2B		
E_4_N_2_H_2_ → E_5_N_2_H_3_	−0.15	1.13
E_5_N_2_H_3_ → E_6_N_2_H_4_	−0.87	0.41
E_6_NH → E_7_NH_2_	0.74	2.02
E_7_NH_2_ → E_8_NH_3_	−0.71	0.57
Without S2B		
E_4_N_2_H_2_′ → E_5_N_2_H_3_′	−0.31	0.97
E_5_N_2_H_3_′ → E_6_N_2_H_4_′	−1.07	0.20
E_6_N_2_H_4_′ → E_7_N_2_H_5_′	1.37	2.65
E_7_NH_2_′ → E_8_NH_3_′	−1.09	0.19

## Data Availability

The data presented in this study are available on request from the corresponding author.

## Supplementary Materials

The following supporting information can be downloaded at: https://www.mdpi.com/article/10.3390/molecules28010065/s1, Figure S1: Structures of the P-cluster and the FeMo cluster, illustrating the QM systems used in the QM/MM geometry optimisations, as well as the names of the nearby residues; Figure S2: Models used for the redox calculations of the P-cluster and the FeMo cluster; Figure S3: Structures used for the E0–E8 states of the FeMo cluster.
