# Supplementary material for: Quantum Mechanical Calculations of Redox Potentials of the Metal Clusters in Nitrogenase
Source: Molecules. 2022 Dec 21;28(1):65. doi: 10.3390/molecules28010065 (PMC9822455; doi:10.3390/molecules28010065)

**Supporting Information**

# Quantum mechanical calculations of redox potentials of the metal clusters in nitrogenase

**Hao Jiang, Oskar K. G. Svensson and Ulf Ryde ***

Department of Theoretical Chemistry, Lund University, Chemical Centre, P. O. Box 124, SE-221 00 Lund, Sweden

Correspondence to Ulf Ryde, E-mail: Ulf.Ryde@teokem.lu.se,

Tel: +46 – 46 2224502, Fax: +46 – 46 2228648

2022-12-20

**Figure S1.** Structures of (a) the P-cluster ($P^N$ resting state) and (b) the FeMo cluster ($E_0$ resting state), illustrating the QM systems used in the QM/MM geometry optimisations, as well as the names of the nearby residues.

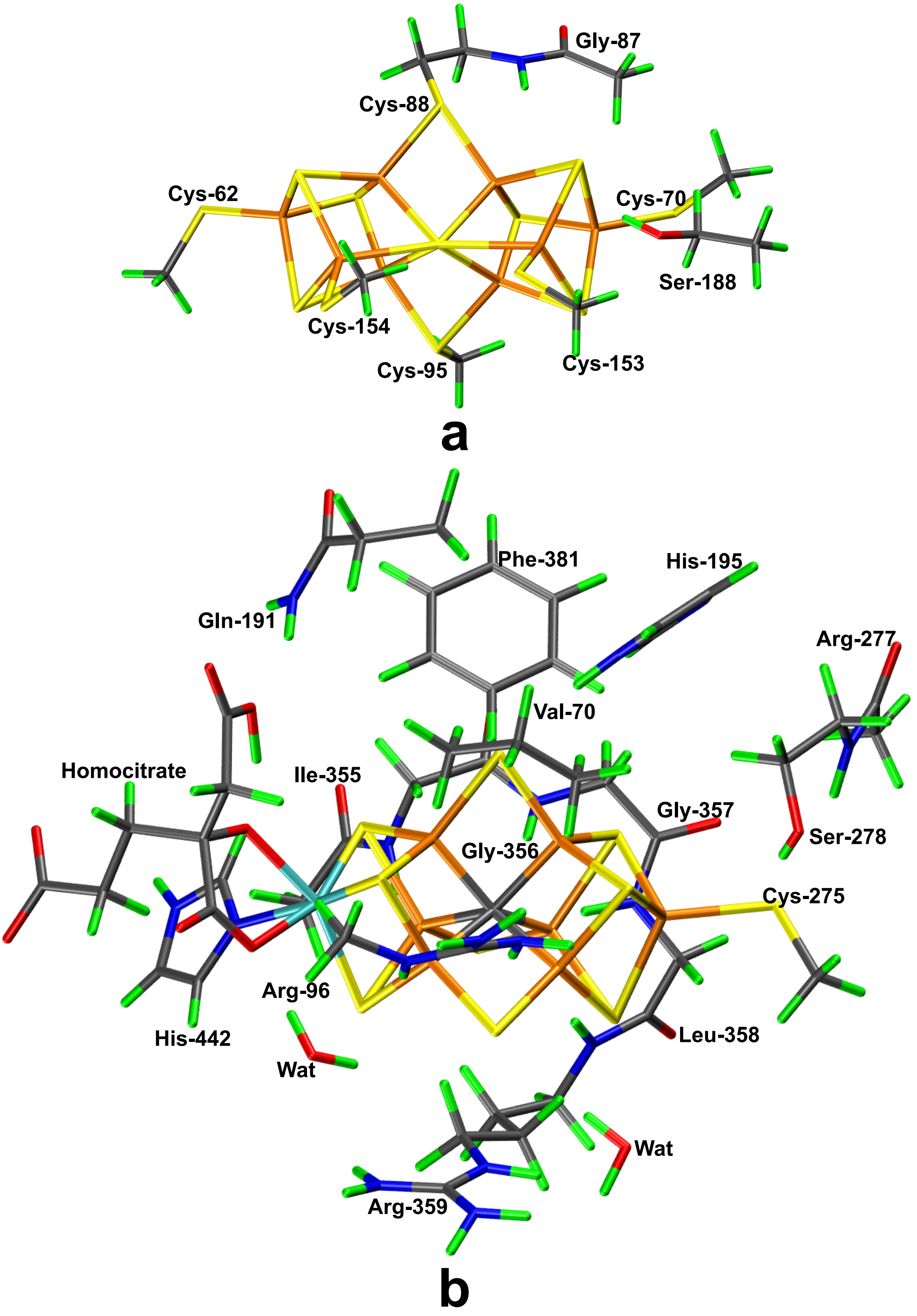

**Figure S2.** Models used for the redox calculations of (a) the P-cluster and (b) the FeMo cluster. The QM system in the QM/MM calculations is shown by a ball and stick model.

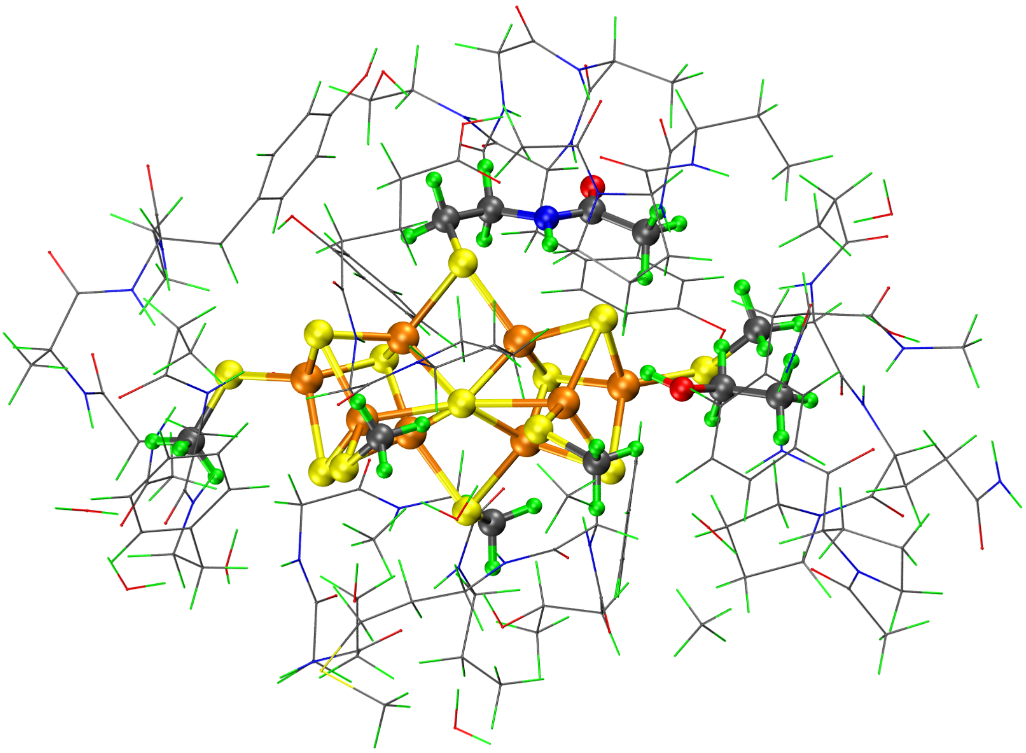

**a**

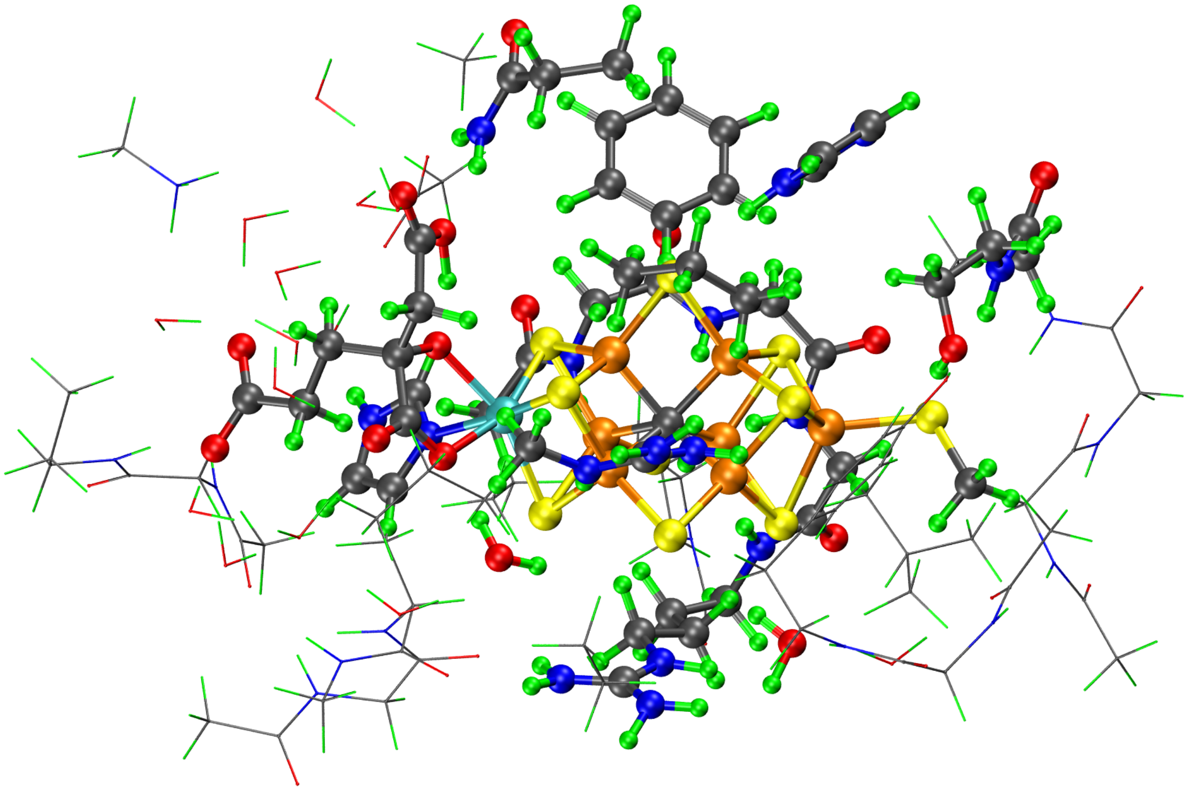

**b**

**Figure S3.** Structures used for the $E_0$–$E_8$ states of the FeMo cluster. The structures are described in Table 5 in the main article.

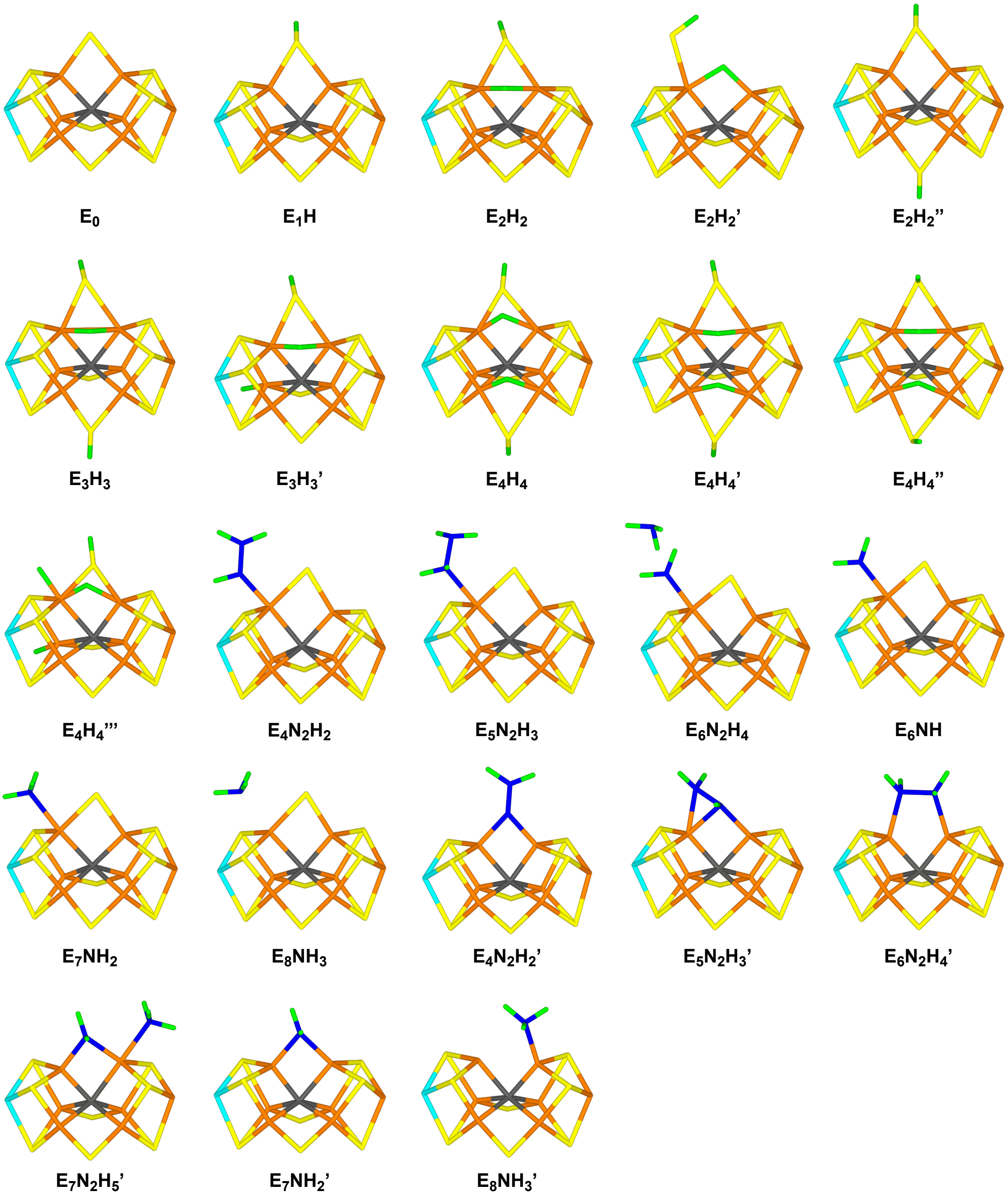

Supplement: Supplementary file 1 [file molecules-28-00065-s001.zip › molecules-2074761-supplementary.pdf]
